# A genome-wide association study identifies a breast cancer risk variant in *ERBB4 *at 2q34: results from the Seoul Breast Cancer Study

**DOI:** 10.1186/bcr3158

**Published:** 2012-03-27

**Authors:** Hyung-cheol Kim, Ji-Young Lee, Hyuna Sung, Ji-Yeob Choi, Sue K Park, Kyoung-Mu Lee, Young Jin Kim, Min Jin Go, Lian Li, Yoon Shin Cho, Miey Park, Dong-Joon Kim, Ji Hee Oh, Jun-Woo Kim, Jae-Pil Jeon, Soon-Young Jeon, Haesook Min, Hyo Mi Kim, Jaekyung Park, Keun-Young Yoo, Dong-Young Noh, Sei-Hyun Ahn, Min Hyuk Lee, Sung-Won Kim, Jong Won Lee, Byeong-Woo Park, Woong-Yang Park, Eun-Hye Kim, Mi Kyung Kim, Wonshik Han, Sang-Ah Lee, Keitaro Matsuo, Chen-Yang Shen, Pei-Ei Wu, Chia-Ni Hsiung, Jong-Young Lee, Hyung-Lae Kim, Bok-Ghee Han, Daehee Kang

**Affiliations:** 1 Center for Genome Science, National Institute of Health, Osong Health Technology Administration Complex, Chungcheongbuk-do, 363-951, Korea; 2 Department of Preventive Medicine, Seoul National University College of Medicine, 103 Daehak-Ro Jongno-Gu, Seoul, 110-799, Korea; 3 Department of Biomedical Sciences, Seoul National University College of Medicine, 103 Daehak-Ro Jongno-Gu, Seoul, 110-799, Korea; 4 Cancer Research Institute, Seoul National University, 103 Daehak-Ro Jongno-Gu, Seoul, 110-799, Korea; 5 Department of Environmental Health, Korea National Open University, 169 Dongsung-Dong, Chongno-Gu, Seoul, 110-791, Korea; 6 Department of Epidemiology and Biostatistics, Tianjin Medical University Cancer Hospital and Institute, Huan-Hu-Xi Road, Ti-Yuan-Bei, He Xi District, Tianjin, 300060, China; 7 Department of Biomedical Science, Hallym University, 1 Hallymdaehak-gill, Chuncheon, Gangwon-do, 200-702, Korea; 8 Department of Surgery, Seoul National University College of Medicine, 103 Daehak-Ro Jongno-Gu, Seoul, 110-799, Korea; 9 Department of Surgery, University of Ulsan College of Medicine and Asan Medical Center, 88 Olympic-Ro 43 gil, Songpa-Gu, Seoul, 138-736, Korea; 10 Department of Surgery, College of Medicine, Soonchunhyang University Hospital, 59 Daesagwan-Ro Yongsan-gu, Seoul, 140-743, Korea; 11 Department of Surgery, Breast and Endocrine Service, Seoul National University Bundang Hospital, 82 Gumi-Ro 173gil, Bundang-Gu, Gyeonggi-do, 463-707, Korea; 12 Department of Surgery, Yonsei University College of Medicine, 50 Yonsei-Ro Seodaemun-Gu, Seoul, 120-752, Korea; 13 Biochemisty and Molecular Biology, Seoul National University College of Medicine, 103 Daehak-Ro Jongno-Gu, Seoul, 110-799, Korea; 14 Carcinogenesis Research Branch, National Cancer Center, 323 Ilsan-Ro, Ilsandong-Gu, Goyang-si, 410-769, Korea; 15 Department of Preventive Medicine, Kangwon National University School of Medicine, 1 Kangwondaehak-gil, Chuncheon-si, Kangwon-do, 200-701, Korea; 16 Division of Epidemiology and Prevention, Aichi Cancer Center Research Institute, 1-1 Kanakoden, Chikusa-ku, Nagoya, 464-8681, Japan; 17 Institute of Biomedical Sciences and Taiwan Biobank, Academia Sinica, 128 Academia Road, Section 2, Nankang, Taipei, 115, Taiwan; 18 College of Public Health, China Medical University, No. 91, Hsueh-Shih Road, Taichung, 40402, Taiwan; 19 Institute of Biomedical Sciences, Academia Sinica, 128 Academia Road, Section 2, Nankang, Taipei, 115, Taiwan

## Abstract

**Introduction:**

Although approximately 25 common genetic susceptibility loci have been identified to be independently associated with breast cancer risk through genome-wide association studies (GWAS), the genetic risk variants reported to date only explain a small fraction of the heritability of breast cancer. Furthermore, GWAS-identified loci were primarily identified in women of European descent.

**Methods:**

To evaluate previously identified loci in Korean women and to identify additional novel breast cancer susceptibility variants, we conducted a three-stage GWAS that included 6,322 cases and 5,897 controls.

**Results:**

In the validation study using Stage I of the 2,273 cases and 2,052 controls, seven GWAS-identified loci [5q11.2/*MAP3K1 *(rs889312 and rs16886165), 5p15.2/*ROPN1L *(rs1092913), 5q12/*MRPS30 *(rs7716600), 6q25.1/*ESR1 *(rs2046210 and rs3734802), 8q24.21 (rs1562430), 10q26.13/*FGFR2 *(rs10736303), and 16q12.1/*TOX3 *(rs4784227 and rs3803662)] were significantly associated with breast cancer risk in Korean women (*P*_trend _< 0.05). To identify additional genetic risk variants, we selected the most promising 17 SNPs in Stage I and replicated these SNPs in 2,052 cases and 2,169 controls (Stage II). Four SNPs were further evaluated in 1,997 cases and 1,676 controls (Stage III). SNP rs13393577 at chromosome 2q34, located in the *Epidermal Growth Factor Receptor 4 *(*ERBB4*) gene, showed a consistent association with breast cancer risk with combined odds ratios (95% CI) of 1.53 (1.37-1.70) (combined *P *for trend = 8.8 × 10^-14^).

**Conclusions:**

This study shows that seven breast cancer susceptibility loci, which were previously identified in European and/or Chinese populations, could be directly replicated in Korean women. Furthermore, this study provides strong evidence implicating rs13393577 at 2q34 as a new risk variant for breast cancer.

## Introduction

Breast cancer, one of the most common malignancies among women worldwide, is a complex polygenic disease in which genetic factors play a significant role in the disease etiology [1,2]. So far, genome-wide association studies (GWASs) have reported over 40 common low-penetrance variants in 25 loci that are associated with the breast cancer risk reported in the National Human Genome Research Institute catalog [3]. The most strongly and consistently associated single-nucleotide polymorphisms (SNPs) reside in intron 2 of the receptor tyrosine kinase *FGFR2 *(rs2981582) at 10q26.13 and near the 5' end of the *TOX3 *gene at 16q12.1 (rs3803662) [4-9]. With the exception of three studies conducted among Asian women [10-12], all other previously published GWASs have been conducted primarily in women of European descent. Several studies, including our study, have investigated previously identified loci in European populations in other ethnic groups and validated the initial findings [13,14]. However, newly discovered loci initially identified in women of European descent tend to be weakly associated with breast cancer in women of Asian descent [10,11] or could not be confirmed in Asians because of the difference in linkage disequilibrium (LD) patterns between ethnic populations, suggesting that additional genetic variants for Asian women remain to be discovered.

In this study, we conducted a three-stage GWAS to identify common breast cancer susceptibility loci and to validate the previously reported loci by using Affymetrix Genome-Wide Human SNP Array 6.0 (Affymetrix, Inc., Santa Clara, CA, USA) with 2,273 patients with breast cancer from the Seoul Breast Cancer Study (SeBCS) and 2,052 healthy controls from a large urban cohort, the Korea Genome Epidemiology Study (KoGES), as stage I. By analyzing data from two replication stages that consisted of 4,049 cases and 3,845 controls, we found strong evidence for a new genetic variant that may be associated with breast cancer risk among Asian women.

## Materials and methods

### Study population

A genome-wide association scan (stage I) was conducted with 2,385 patients with breast cancer from the SeBCS and 2,392 healthy controls from a large urban cohort that is participating in the KoGES (Supplementary methods in Additional file 1). For stage II, 2,052 cases were selected among the patients who were the participants in SeBCS but not included in stage I and 2,169 controls from another cohort recruited from two small cities with both urban and rural areas as part of KoGES. For stage III, 1,997 cases were selected from two independent breast cancer studies - the Korean Hereditary Breast Cancer (KOHBRA) study (*n *= 1,289) and the Yonsei Breast Cancer Study (*n *= 708) - and 1,676 controls were selected from health examinees from rural populations to study the risk factors for chronic diseases. Detailed descriptions of these participants are provided in Supplementary methods in Additional file 1, and descriptive statistics of the study subjects are shown in Table S2 of Additional file 2. The study protocols were approved by the institutional review boards of Seoul National University Hospital (institutional review board # H-0503-144-004) and each collaborating institute (Description of study participants in Additional file 1). Informed consent was obtained from all participants.

### Genotyping

A GWAS (stage I) was performed by a single platform by using the Affymetrix Genome-Wide Human SNP Array 6.0 chip (Affymetrix, Inc.). In total, 4,777 samples were genotyped by using 500 ng of genomic DNA from peripheral blood. The Birdseed V2 algorithm was used to call the genotypes [15].

In total, 30 quality control (QC) samples were genotyped by using Affymetrix SNP Array 6.0. The average concordance rate between the QC samples was 99.8%. For internal validation of the Affymetrix SNP Array 6.0 platform, 12 SNPs were genotyped for all subjects by SNPstream UHT (12-plex, SNP-IT assay, Orchid Biosciences, Princeton, NJ). Samples of subjects that had a genotype call rate of below 95%, a high heterozygosity rate, or an incorrectly imputed gender were excluded. Calculated genome-wide average identity by state (IBS) between each pair of individuals was used to identify individuals who appeared to be in relationships with first-degree relatives or in relationships with more distant relatives whose clusters were tightly linked to the first-degree relatives. Pairwise IBS between individuals was calculated by using a subset of pruned markers (74,965 SNPs) that are in approximate linkage equilibrium. IBS analysis was performed by using the PLINK software package. Multidimensional scaling analyses based on pairwise IBS showed that, apart from some outliers, all subjects clustered closely with HapMap Asians (Figure S2 of Additional file 3). Subjects with a cancer history and patients with diagnosed benign breast cancer were subsequently excluded. Finally, 4,325 individuals remained in the association analyses (Table S1 of Additional file 2).

To ensure quality data for SNPs, SNPs were excluded if they met any of the following QC criteria: (a) deviation from the Hardy-Weinberg equilibrium *P *value of less than 10^-6^, (b) a genotype call rate of less than 95%, (c) a minor allele frequency (MAF) of less than 1%, (d) a poor cluster plot, (e) filtering out differential missingness between cases and controls (*P *< 10^-4^), and (f) multiple positioning or mitochondrial SNPs or both. In total, 555,525 Affymetrix SNP Array 6.0 SNPs were used for the final association analyses (Table S1 of Additional file 2).

Genotyping for stages II and III was performed by using the 5' exonucleaseassay (TaqMan) employing the ABI Prism 7900HT Sequence Detection System (Applied Biosystems, Foster City, CA) in accordance with the instructions of the manufacturer. Primers and probes were supplied directly by Applied Biosystems (Foster City, CA, USA) as Assays-By-Design. For QC, about 2.2% of the samples were genotyped repeatedly. Only those SNPs that satisfied a concordance rate of greater than 99% in duplicates and a genotype success rate of greater than 99% were included in the subsequent replication phases.

### Single-nucleotide polymorphism selection for validation study using stage I

We selected the SNPs if they were implicated in previous GWASs and reported from the National Human Genome Research Institute catalog [16]. We included SNPs only if they had an assigned reference allele, a defined MAF, and an estimated odds ratio (OR) or a beta-coefficient and a 95% confidence interval (CI). For the SNPs that were not genotyped by using Affymetrix SNP Array 6.0 or successfully imputed (imputation QC r^2 ^was less than 0.3), we selected the best tagging SNPs on the basis of the LD metrics (r^2 ^and D'). Thus, we selected rs10736303 for the SNPs at 10q21.13 (rs1219648, rs2981572, rs2981585, and rs2981579) and rs10483813 for 14q24.1/*RAD51L1 *rs999737. We did not include the SNP rs614367 at 11q13.3/*MYEOV, CCND1, ORAOV1, FGF19, FGF4, FGF3 *since the MAF of rs614367 is very low in this study (MAF = 0.002) [9].

### Single-nucleotide polymorphism selection for replication in stage II

After stage I, SNPs for replication were selected on the basis of the following criteria among the 555,525 SNPs that were directly genotyped and that had passed the QC procedure: SNPs (a) with an MAF of more than 5% for either cases or controls, (b) with very clear genotyping clusters, (c) with a *P*_trend _of per-allele OR of not more than 5 × 10^-4^, and (d) not in strong LD (r^2 ^< 0.5) with any of the GWAS-identified risk variants. Additionally, to select the SNPs resided in SNP cluster we selected the loci within which at least two SNPs had a *P*_trend _of per-allele OR of not more than 5 × 10^-3^. When multiple SNPs showed LD within 100 kb (r^2 ^> 0.2), the SNP with the lowest *P*_trend _was selected for replication.

### Single-nucleotide polymorphism imputation

To infer the genotype of SNPs that were not observed in the Affymetrix Genome-Wide Human SNP Array 6.0 used in the present study, SNP imputation was carried out by using the hidden Markov model as implemented in MACH 1.0 [17,18]. Imputation was based on 555,525 autosomal SNPs that were genotyped in stage I and that had passed the QC procedure, and the phased CHB + JPT data from HapMap Phase II (release 22) were used as the reference panel, which consisted of over 2.4 million SNPs. In total, 2,210,823 SNPs showed an imputation quality score (r^2^) of at least 0.30. The average r^2 ^of SNPs not found on the array but included in the validation study (15 SNPs in Table 1) is 0.97.

**Table 1:** Association of previously identified loci with breast cancer risk in 2,257 cases and 2,052 controls in the Seoul Breast Cancer Study

Region/Reported gene(s)	SNP	Loci evaluated in this study					Loci reported in genome-wide association studies				
		**Risk allele**	**RAF**	**OR (95% CI)^a^**	***P***	**Genotyping**	**Risk allele**	**RAF**	**OR (95% CI)**	***P***	**Reference**

1p11.2/*NOTCH2, FCGR1B*	rs11249433	C	0.04	1.11 (0.88-1.40)	3.8 × 10^-1^	Imputed	C	0.39	1.16 (1.09-1.24)	7 × 10^-10^	[8]

2q35/*Intergenic*	rs13387042	A	0.10	1.11 (0.96-1.28)	1.6 × 10^-1^	Imputed	A	0.50	1.2 (1.14-1.26)	1 × 10^-13^	[6]

3p24.1/*SLC4A7*	rs4973768	T	0.22	1.09 (0.98-1.21)	1.2 × 10^-1^	Imputed	T	0.47	1.16 (1.10-1.24)	6 × 10^-7^	[9]

5q11.2/*MAP3K1*	rs16886165	G	0.35	1.14 (1.04-1.25)	6.5 × 10^-3^	Imputed	G	0.15	1.23 (1.12-1.35)	5 × 10^-7^	[8]

	rs889312	C	0.55	1.16 (1.06-1.27)	8.5 × 10^-4^	Imputed	C	0.28	1.13 (1.10-1.16)	7 × 10^-20^	[5]

5p12/*MRPS30*	rs7716600	A	0.48	1.13 (1.03-1.23)	6.9 × 10^-3^	Imputed	A	0.23	1.24 (1.14-1.34)	7 × 10^-7^	[34]

	rs4415084	T	0.56	1.09 (0.99-1.19)	7.8 × 10^-2^	Imputed	T	0.42	1.17 (1.11-1.22)	8 × 10^-11^	[24]

5p15.2/*ROPN1L*	rs1092913	G	0.30	1.11 (1.01-1.22)	2.8 × 10^-2^	Imputed	T	0.13	1.45 (1.24-1.69)	2 × 10^-6^	[35]

6q22.33/*ECHDC1, RNF146*	rs2180341	G	0.27	1.03 (0.93-1.13)	6.0 × 10^-1^	Typed	G	0.21	1.41 (1.25-1.59)	3 × 10^-8^	[36]

6q25.1/*ESR1, C6orf97*	rs3734805	C	0.33	1.20 (1.09-1.33)	1.8 × 10^-4^	Imputed	C	0.08	1.19 (1.11-1.27)	1 × 10^-7^	[24]

	rs2046210	A	0.35	1.29 (1.18-1.41)	5.8 × 10^-8^	Typed	A	0.37	1.29 (1.21-1.37)	2 × 10^-15^	[10]

7q32.3/*NR*	rs2048672	C	0.49	1.05 (0.97-1.15)	2.3 × 10^-1^	Typed	C	0.45	1.11 (1.05-1.17)	6 × 10^-6^	[12]

8q24.21/*Intergenic*	rs13281615	C	0.57	1.04 (0.95-1.14)	3.8 × 10^-1^	Imputed	C	0.40	1.08 (1.05-1.11)	5 × 10^-12^	[5]

	rs1562430	T	0.87	1.16 (1.01-1.33)	3.0 × 10^-2^	Imputed	T	0.58	1.17 (1.10-1.25)	6 × 10^-7^	[9]

9p21.3/*CDKN2A, CDKN2B*	rs1011970	T	0.07	1.00 (0.84-1.19)	9.9 × 10^-1^	Imputed	T	0.17	1.09 (1.04-1.14)	3 × 10^-8^	[9]

9q31.2/*KLF4, RAD23B, ACTL7A*	rs865686	T	0.93	1.02 (0.86-1.21)	8.1 × 10^-1^	Imputed	T	0.61	1.12 (1.09-1.18)	2 × 10^-10^	[24]

10p15.1/*ANKRD16, FBXO18*	rs2380205	C	0.91	1.11 (0.95-1.29)	1.8 × 10^-1^	Typed	C	0.57	1.06 (1.02-1.10)	5 × 10^-7^	[9]

10q21.2/*ZNF365*	rs10995190	G	0.98	1.02 (0.76-1.38)	8.9 × 10^-1^	Imputed	G	0.85	1.16 (1.10-1.22)	5 × 10^-15^	[9]

	rs10822013	T	0.47	1.06 (0.97-1.15)	2.1 × 10^-1^	Typed	T	0.47	1.12 (1.06-1.18)	6 × 10^-9^	[12]

10q22.3/*ZMIZ1*	rs704010	A	0.29	1.08 (0.98-1.19)	1.2 × 10^-1^	Typed	A	0.39	1.07 (1.03-1.11)	4 × 10^-9^	[9]

10q26.13/*FGFR2*	rs10510102	G	0.18	1.01 (0.90-1.13)	8.4 × 10^-1^	Typed	G	0.17	1.12 (1.07-1.17)	2 × 10^-6^	[24]

	rs10736303^b^	G	0.53	1.17 (1.07-1.28)	4.4 × 10^-4^	Typed	T	0.41	1.17 (1.07-1.27)	2 × 10^-10^	[8]

11p15.5/*LSP1*	rs3817198	C	0.16	1.00 (0.89-1.13)	9.5 × 10^-1^	Typed	C	0.30	1.07 (1.04-1.11)	3 × 10^-9^	[5]

14q24.1/*RAD51L1*	rs10483813^c^	T	0.97	1.21 (0.93-1.56)	1.6 × 10^-1^	Imputed	C	0.76	1.06 (1.01-1.14)	2 × 10^-7^	[8]

16q12.1/*TOX3*	rs4784227	T	0.29	1.27 (1.15-1.40)	1.5 × 10^-6^	Typed	T	0.24	1.24 (1.20-1.29)	1 × 10^-28^	[11]

	rs3803662	T	0.64	1.24 (1.14-1.36)	2.4 × 10^-6^	Typed	T	0.27	1.28 (1.21-1.35)	6 × 10^-19^	[5]

19q13.41/*ZNF577*	rs10411161	C	0.72	1.01 (0.92-1.11)	8.6 × 10^-1^	Typed	T	0.13	1.42 (1.22-1.65)	7 × 10^-6^	[35]

The average call rate was 99.8% (range of 99.4% to 100%) for typed single-nucleotide polymorphisms (SNPs), and the average of imputation quality score from MACH (r^2^) was 0.97 (range of 0.95 to 1.00). ^a^Per-allele odds ratio (OR) adjusted for age; ^b^proxy SNP of rs2981579 (r^2 ^= 0.48 in CHB+JPT; r^2 ^= 0.74 in CEU); ^c^proxy SNP of rs999737 (r^2 ^= 0.50 in CHB+JPT; r^2 ^= 0.98 in CEU). CEU, CEPH Utah residents with ancestry from Northern and Western Europe; CHB+JPT, Han Chinese from Beijing + Japanese from Tokyo; CI, confidence interval; RAF, risk allele frequency.

### Bioinformatics

The LD metrics (r^2 ^and D') between SNPs were calculated by using Haploview version 4.2 software (Whitehead Institute, Cambridge, MA, USA) based on release 27, NCBI Build 36. Regional plots were drawn by using LocusZoom standalone version 1.1 [19] based on HapMap Phase II JPT+CHB for all SNPs in Figure S3 of Additional file 3 except for rs10736303, which was based on 1000 Genome August 2009 JTP+CHB.

### Statistical analyses

Association on breast cancer risk was estimated by ORs and 95% CIs while assuming an additive model by logistic regression analysis adjusted for age. GWAS stage (stage 1) analyses were conducted primarily by using PLINK program version 1.06 [20] for directly genotyped SNPs, and the MACH2dat program [17,18] was used for imputed SNPs. For the comparison of the observed and expected distribution of test statistics, quantile-quantile analysis of 2-degrees-of-freedom logistic regression statistics was applied. The genomic inflation factor lamda (λ) was calculated as 1.043, suggesting that the population substructure, if any, should not have a substantial effect on result (Figure S1 of Additional file 3).

To control the risk of false discoveries, the multiple-comparison-adjusted *P *values for stage II, stage III, and combined analysis were calculated by the Benjamini-Hochberg false discovery rate method [21]. Cochran's Q statistic to test for heterogeneity and the I^2 ^statistic to quantify the proportion of the total variation due to heterogeneity across all stages were calculated. Combined statistics were estimated from meta-analysis while assuming a fixed-effects model since there was no evidence of heterogeneity. To assess the relationship according to estrogen receptor (ER) or progesterone receptor (PR) status or both, ORs and 95% CIs were estimated after stratification by hormone receptor status. Case-only *P *value was used to test for heterogeneity and was estimated by using a polytomous logistic regression model with receptor status as the outcome variables.

The statistical power of detecting the ORs reported in previous GWASs was calculated by using Quanto version 1.2.4. All other analyses other than GWAS stage were done by using SAS version 9.2 (SAS Institute Inc., Cary, NC, USA) and STATA version 11.2 (StataCorp LP, College Station, TX, USA).

## Results

### Validation of previously identified breast cancer susceptibility loci in Korean women

**Figure 1:**
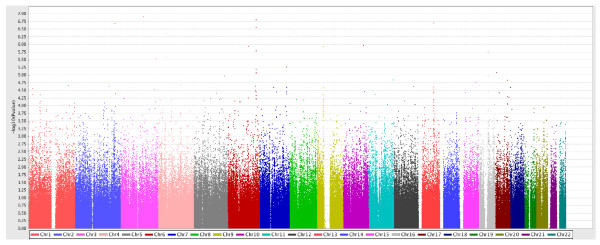
**Manhattan plot for 555,525 genotyped single-nucleotide polymorphisms in 2,273 cases and 2,052 controls**.

We found that multiple genomic locations were potentially associated with the risk of breast cancer (Figure 1). Table 1 presents the test of associations between the previously reported loci and breast cancer risk in 2,257 cases and 2,052 controls. Among the 27 SNPs reported in published GWASs, 10 SNPs located in seven loci showed significant associations with breast cancer risk (*P*_trend _< 0.05). Plots of regional LD and strength of association by chromosomal position for these seven loci of interest are shown in Figure S3 of Additional file 3.

The strongest and most significant association was observed in 6q25.1/*ESR1 *rs2046210 (OR = 1.29; 95% CI = 1.18 to 1.41; *P*_trend _= 5.84 × 10^-8^) followed by 16q12.1/*TOX3 *rs4784227 (OR = 1.27; 95% CI = 1.15 to 1.40; *P*_trend _= 1.51 × 10^-6^), and these values are slightly similar to the magnitude and direction of previous reports. The SNPs 6q25.1/*ESR1 *rs3734802 and 16q12.1/*TOX3 *rs3803662, which had a moderate LD with rs2046210 (r^2 ^= 0.317 in CHB+JPT) and rs4784227 (r^2 ^= 0.139 in CHB+JPT), respectively, were also significantly associated with breast cancer risk (OR = 1.20; 95% CI = 1.09 to 1.33; *P*_trend _= 1.8 × 10^-4 ^and OR = 1.24; 95% CI = 1.14 to 1.36; *P*_trend _= 2.41 × 10^-6^, respectively). Although allele frequencies of 5q11.2/*MAP3K1 *rs889312 (like 16q12.1/*TOX3 *rs3803662) were substantially different between Europeans and Koreans, the rs889312 C allele was also significantly associated with increased risk of breast cancer (OR = 1.16; 95% CI = 1.06 to 1.27; *P*_trend _= 8.49 × 10^-4^). The 10q26.13/*FGFR2 *rs10736303 (proxy of rs2981579), 5p15.2/*ROPN1L *rs1092913, 5q12/*MRPS30 *rs7716600, and 8q24.21 rs1562430 were also confirmed to be associated with breast cancer risk, although the magnitude of the last three SNPs was smaller than that of previous reports. However, 14q24.1/*RAD51L1 *rs10483813 (proxy of rs999737) showed no significant association with an OR of 1.21. Additionally, none of the remaining 17 SNPs was replicated in our study (*P *> 0.05), and the effect sizes were estimated to be less than 1.10, which is lower than that of the estimated ones in women of European descent.

We further assessed these associations according to ER and PR status (Table S3 of Additional file 2). The 5q12/*MRPS30 *rs7716600 and rs4415084 exhibited a stronger association with ER^+ ^than with ER^- ^tumors (*P*_heterogeneity _= 0.02 and *P*_heterogeneity _= 0.05, respectively). Although no overall associations were found for 2q35 rs13387042 and 10p15.1/*ANKRD16, FBXO18 *rs2380205, stratified analysis revealed a statistically significant association with ER^+ ^(*P*_trend _= 0.03 and *P*_trend _= 0.04) or PR^+ ^(*P*_trend _= 0.05 and *P*_trend _= 0.05) tumors but not with ER^- ^or PR^- ^tumors. The test for heterogeneity was significant only for 10p15.1/*ANKRD16, FBXO18 *rs2380205 (*P*_heterogeneity ER+ vs. ER- _= 0.030 and *P*_heterogeneity PR+ vs. PR- _= 0.040). Additionally, two SNPs at 6q25.1/*ESR1 *(rs2046210 and rs3784805) exhibited a stronger association with ER^- ^than with ER^+ ^tumors, although the differences were not statistically significant. There were no differences in associations by ER or PR status for the remaining SNPs.

### Analysis of 2q34 rs13393577 and breast cancer risk

**Figure 2:**
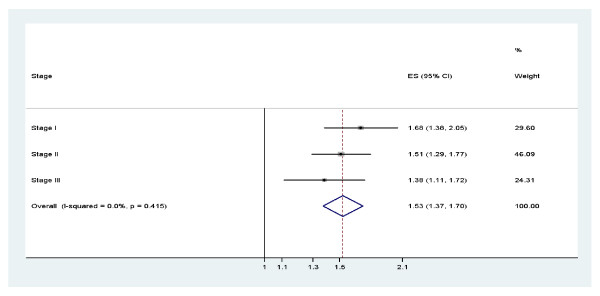
**Forest plot**. Result of pooled analysis of rs13393577 on the basis of estimated per-allele odds ratio from each stage. CI, confidence interval; ES, effect size.

**Figure 3:**
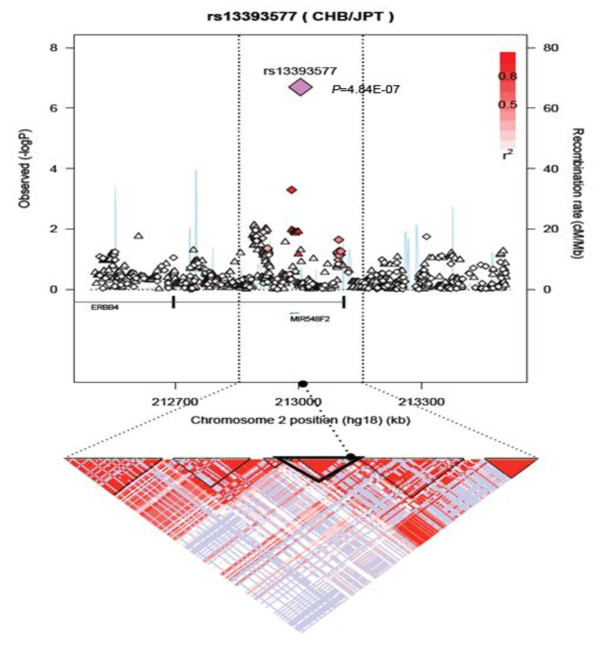
**Regional association plot of the 2q34 (rs13393577) locus**. The results of association signals (-log P) are shown for directly genotyped (diamonds) and imputed (triangle) single-nucleotide polymorphisms (SNPs) distributed in a genomic region 500 kb to either side of rs1339577. Red reflects the linkage disequilibrium (r^2^) with the top SNP, and increasing red hue is associated with increasing r^2^. The blue bars show the recombination rate based on HapMap phase II release 22 JPT and CHB populations. The bottom panels illustrate the locations of known genes. The genomic position is based on the UCSC (University of California at Santa Cruz) Genome Browser assembly, March 2010. CHB, Han Chinese from Beijing; JPT, Japanese from Tokyo.

To search for additional independent genetic risk variants in Korean women, we selected the most suggestive 17 SNPs from stage I and genotyped in an independent set of 2,052 cases and 2,169 controls (stage II). Among the 17 SNPs evaluated in stage II, only one SNP, rs13393577 at 2q34, was significantly associated with breast cancer risk. The estimated ORs for rs13393577 were as follows: OR_heterozygote _= 1.39 (95% CI = 1.16 to 1.67; *P *= 2.05 × 10^-5^), OR_homozygote _= 5.57 (95% CI = 2.26 to 13.7; *P *= 4.10 × 10^-3^), and OR_per-allele _= 1.51 (95% CI = 1.29 to 1.78; *P*_trend _= 1.1 × 10^-5^). SNP rs13393577 and three more SNPs (3q26.32 rs3806685, 6q25.1 rs9498283, and 17q24.3 rs11077488) showing marginally significant associations in stage II (*P*_trend _< 0.10) were further evaluated in stage III, which included 1,997 cases and 1,676 controls. Again, rs13393577 showed a significant association with breast cancer risk (Table 2). The *P*_trend _reached 8.8 × 10^-14 ^in the combined analysis (Figure 2). The SNP rs3806685 at 3q26.32 was also associated with breast cancer risk with an OR_per-allele _of 1.18 (95% CI = 1.04 to 1.34; *P*_trend _= 1.8 × 10^-2^) in stage III; however, the combined OR was not significant (*P*_trend _= 5.9 × 10^-1^). The other two SNPs showed no significant association in stage III or in the combined analysis (*P *> 0.05). The test for heterogeneity suggested no difference in the genetic effects across ER or PR status for rs13393577 (data not shown). To capture additional signals for rs13393577, we investigated the SNPs nearby rs13393577 (Figure 3). Among the directly genotyped SNPs, two SNPs are in strong LD (r^2 ^> 0.8) with rs13393577, and the smallest *P *values were 1.2 × 10^-3 ^for rs6756468 (r^2 ^= 1.00) and 2.6 × 10^-2 ^for rs16848753 (r^2 ^= 0.81). SNP rs6756468 is in tight LD with rs13393577 located in the LD block containing the *mir-548f-2 *gene. Several imputed SNPs in high LD with rs13393577 were nominally associated with breast cancer risk (*P *< 0.05) (Table S5 of Additional file 2).

**Table 2:** Results of four single-nucleotide polymorphisms and breast cancer risk identified in genome-wide association studies (stage I) and replication stages (stages II and III)

SNP^a^	Genomic location^b^	Stage	Number of cases	Number of controls	MAF in cases	MAF in controls	HWE *P*	Heterozygote OR (95% CI)^a^	Homozygote OR (95% CI)^a^	Per-allele OR (95% CI)^c^	*P*_trend_^d^
rs13393577	213,005,108	I	2,269	1,992	0.066	0.039	0.25	1.72 (1.38-2.13)	2.25 (0.79-6.42)	1.68 (1.38-2.06)	4.8 × 10^-7^

(C/T)	(2q34)	II	2,037	2,166	0.096	0.066	0.22	1.39 (1.16-1.67)	5.57 (2.26-13.7)	1.51 (1.29-1.78)	1.1 × 10^-5^

		III	1,952	1,644	0.061	0.047	0.83	1.35 (1.07-1.70)	2.70 (0.81-9.03)	1.38 (1.11-1.71)	1.3 × 10^-2^

		Combined	6,258	5,802	0.074	0.051		1.47 (1.28-1.70)	3.49 (1.93-6.33)	1.53 (1.37-1.70)	8.8 × 10^-14^

rs9498283	149,646,875	I	2,266	2,051	0.451	0.500	0.08	0.79 (0.68-0.91)	0.67 (0.56-0.79)	0.81 (0.75-0.89)	5.3 × 10^-6^

(A/G)	(6q25.1)	II	2,049	2,145	0.449	0.470	0.05	0.97 (0.84-1.12)	0.85 (0.71-1.01)	0.92 (0.85-1.01)	6.8 × 10^-1^

		III	1,988	1,657	0.453	0.478	0.28	1.02 (0.87-1.19)	0.85 (0.70-1.03)	0.93 (0.84-1.02)	1.6 × 10^-1^

		Combined	6,303	5,853	0.451	0.451		0.92 (0.79-1.07)	0.78 (0.66-0.92)	0.89 (0.82-0.96)	9.6 × 10^-3^

rs11077488	65,801,677	I	2,273	2,052	0.150	0.185	0.58	0.77 (0.67-0.89)	0.60 (0.41-0.86)	0.77 (0.69-0.87)	1.2 × 10^-5^

(C/T)	(17q24.3)	II	2,046	2,141	0.155	0.171	0.93	0.88 (0.76-1.01)	0.87 (0.60-1.27)	0.90 (0.80-1.01)	9.7 × 10^-1^

		III	1,984	1,669	0.164	0.163	0.90	1.02 (0.87-1.18)	0.98 (0.65-1.49)	1.01 (0.89-1.15)	1.1 × 10^-1^

		Combined	6,303	5,862	0.156	0.174		0.88 (0.76-1.02)	0.79 (0.59-1.07)	0.89 (0.76-1.03)	1.6 × 10^-1^

rs3806685	180,522,445	I	2,262	2,051	0.156	0.189	0.95	0.90 (0.79-1.03)	0.25 (0.15-0.41)	0.78 (0.69-0.88)	3.4 × 10^-5^

(A/G)	(3q26.32)	II	2,040	2,163	0.169	0.177	0.11	0.84 (0.73-0.97)	1.04 (0.72-1.52)	0.90 (0.80-1.01)	9.7 × 10^-1^

		III	1,971	1,657	0.189	0.162	0.93	1.22 (1.05-1.42)	1.24 (0.83-1.83)	1.18 (1.04-1.34)	1.8 × 10^-2^

		Combined	6,273	5,871	0.171	0.177		0.97 (0.78-1.21)	0.69 (0.28-1.70)	0.94 (0.74-1.18)	5.9 × 10^-1^

^a^Major/Minor allele; ^b^location is based on NCBI Build 36; ^c^odds ratio (OR) adjusted for age; ^d^*P*_trend _values of stage II, stage III, and combined analysis were adjusted for the multiple comparisons by using false discovery rate. CI, confidence interval; GWAS, genome-wide association study; HWE, Hardy-Weinberg equilibrium; MAF, minor allele frequency; SNP, single-nucleotide polymorphism.

## Discussion

In the present study, we conducted a three-stage GWAS in Korean women (6,322 cases and 5,897 controls). We not only confirmed previously identified loci in Europeans or Chinese populations or both but also found rs13393577 at 2q34/*ERBB4 *as a new breast cancer susceptibility variant in Korean women.

In the validation study, we evaluated whether 27 SNPs in the 20 GWAS-identified loci were also relevant in our population using stage I and identified that 10 SNPs at seven loci were significantly associated with breast cancer risk. As anticipated, the strongest and the most significant results were observed in rs2046210 at 6q25.1/*ESR1 *and rs4784227 at 16q12.1/*TOX3*, and these results are slightly similar to those of the magnitude and direction of previous reports conducted in a Chinese population [10,11]. For the SNPs rs2048671 at 7q32.3/*NR *and rs10822013 at 10q21.2/*ZNF365*, which were also identified in Asians, we recently reported significant associations with breast cancer risk through multi-stage GWAS with a cumulative sample size up to over 34,000 East Asian subjects (OR _per-allele _= 1.10; 95% CI = 1.07 to 1.14; *P*_trend _= 5.87 × 10^-9 ^and OR = 1.08; 95% CI = 1.04 to 1.11; *P*_trend _= 6.21 × 10^-6^) [12]. However, the associations of these SNPs were not significant in this study, possibly because of its limited power.

Among the remaining 23 SNPs that were initially identified in Europeans, eight SNPs - rs10736303 (10q26.13/*FGFR2*, proxy of rs2981579), rs3803662 (16q12.1/*TOX3*), rs7716600 (5p12/*MRPS30*), rs16886165 and rs889312 (5q11.2/*MAP3K1*), rs3734805 (6q25.1/*ESR1*), and rs1562430 (8q24.21) - showed significant associations in the same direction except for rs1092913 (5p15.2/*ROPN1L*) with the G allele as the risk allele. The effect sizes of the confirmed variants were similar to or smaller than those of the initially identified ones. This phenomenon has been frequently observed in validation studies using ethnic populations different from the population used for the initial findings [22,23].

In addition, we could not evaluate the SNPs (rs1219648, rs2981572, rs2981585, and rs2981579) previously identified within intron 2 of *FGFR2 *at 10q21.13, because they were not genotyped or successfully imputed (imputation QC r^2 ^< 0.3). Thus, we selected rs10736303 as the best tagging SNP capturing 10q26.13/*FGFR2 *since it is in high LD with the reported SNPs with pairwise r^2 ^values of 0.67 for rs2981579 (r^2 ^= 0.48 in CHB+JTP; r^2 ^= 0.74 in CEU), 0.57 for rs2981575 (r^2 ^= 0.36 in CHB+JPT; r^2 ^= 0.72 in CEU), and 0.53 for rs1219648 (r^2 ^= 0.29 in CHB+JPT; r^2 ^= 0.72 in CEU) on the basis of our data. Furthermore, the rs10736393 is located at intron 2 of *FGFR2 *within the sequences conserved across all placental mammals and suggested to be a functional variant to regulate *FGFR *expression by generating a putative ER-binding site [5]. In the present study, the rs10736393 G allele was significantly associated with increased breast cancer risk with an effect size of rs2981579 that was the same as in a previous report [8]. However, the recently added SNP, rs10510102, located in the 300-kb telomeric region of intron 2 of *FGFR2 *but not with a genome-wide significance level (*P *= 1.6 × 10^-6^), was not replicated in the present study [24].

Subgroup analysis revealed that some of the validated associations differed by ER or PR status. Recent studies showed stronger associations with ER^+ ^than with ER^- ^tumors for several loci - rs13387042(2q35), rs4973768 (3p24), rs889312 (5q11.2/*MAP3K1*), rs7716600 (5q12/*MRP30*), rs13281615 (8q24), rs1219648 and rs2981582 (10q26.13/*FGFR2*), and rs3803662 (16q12) - and with PR^+ ^than with PR^- ^tumors for rs2981582 (10q26.13/*FGFR2*) [6,25,26]. Among these loci, rs13387042 (2q35), rs7716600, and rs4415084 (5q12/*MRP30*) showed significantly different associations by ER status, and rs4973768 (3p24) also showed a stronger association with ER^+ ^tumors, although the test for heterogeneity was not significant. The association of rs2380205 (10p15.1/*ANKRD16, FBXO18*) with breast cancer risk was also stronger for ER^+ ^or PR^+ ^tumors than with negative tumors, and this heterogeneity in association remains to be evaluated in other populations.

The stronger association of rs2046210 at 6q25.1/*ESR1 *with ER^- ^than with ER^+ ^tumors has been well documented [10]. In the present study, we could also observe this heterogeneity for rs2046210 and its nearby SNP rs3784805, although the differences were not statistically significant. Direct replication in some of the loci showing significant differences in associations according to ER or PR status provides further support for the hypothesis that intrinsic subtypes of breast cancer should have different etiologic pathways; thus, the polygenic component of these subtypes of breast cancer should be different [27].

There are several potential reasons for the failure of validation for previously identified loci in women of European descent. First, several risk variants could escape detection because of the limited statistical power caused by either low allele frequency or a very small effect size of the initial findings. There are several SNPs of which the allele frequencies in Koreans are substantially lower than in Europeans: SNP rs11249433 at 1p11.2/*NOTCH2, FCGR1B *(4% versus 39%), rs1011970 at 9p21.3/*CDKN2A, CDKN2B *(7% versus 17%), rs865686 at 9q31.2/*KLF4, RAD23B, ACTL7A *(7% versus 24%), rs10995190 at 10q21.2/*ZNF365 *(2% versus 15%), and rs10483813, proxy of rs999737, at 14q24.1/*RAD51L *(3% versus 24%). Thus, we have only 8% to 30% of the statistical power to detect the reported effect sizes of 1.06 to 1.16 for these SNPs with the current sample size. We could not exclude the possibility that the effect size of the original reports could be represented as exaggerated ORs caused by 'winner's curse'. Second, a difference in underlying genomic structure between ethnicities could produce the bias to cover SNPs tagging the causal variants, although the reported SNPs could work effectively in women of European descent. Another possibility is that some of the variants evaluated may not be strongly associated with breast cancer risk in Asian women such as shown in the null association of rs2180341 (6q22.33). For rs3180341, we had a statistical power of 80% to detect an OR as small as 1.15; furthermore, the lack of an association has been shown in a study conducted in a Chinese population [13]. Moreover, the risk profiles of genetic variants could be manifested differently in different ethnic populations, assuming that the relative contribution of the risk variants to carcinogenic pathways of breast cancer varies between different populations. Finally, the interactions of environmental exposures, lifestyle, or other effect modifiers and even the difference in breast cancer prevalence could have an effect on the penetrance of these alleles.

The *ERBB4*, harboring rs13393577 in the first intron at 2q34, is a member of the epidermal growth factor (*EGF/ERBB*) family of receptor tyrosine kinases, which are key activators of signaling pathways involved in cell division, migration, adhesion, differentiation, and apoptosis [28]. It is reported that *ERBB4 *is frequently overexpressed in breast cancer, and the expression of transcripts encoding the cleavable *ERBB4 *isoforms was associated with ER expression and a high histological grade of differentiation [29]. Rokavec and colleagues [30] identified the presence of five germ-line variants in the *ERBB4 *5'-untranslated region and reported that one of these variants (*ERBB4 *-782T > G) was associated with breast cancer risk from the different promoter activity according to the different allele. However, rs13393577 is not in LD with *ERBB4 *-782T > G; thus, the potential influence of rs13393577 is unlikely to be mediated through this previously reported variant.

We conducted an *in silico *functional analysis to assess the potential biological function of rs13393577. The rs13393577 C allele had no predicted binding site, whereas several transcription factors were predicted to bind the rs13393577 T allele implementing six high-scoring binding sites (maximum score = 92.7 points; minimum score = 85.9 points) [31]. In agreement with this, FASTSNP scored rs13393577 as 1-2 (intronic enhancer) [32]. Additionally, Murabito and colleagues [33] have shown that three SNPs in *ERBB4 *(rs905883, rs7564590, and rs7558615) were associated with breast cancer risk in a family-based GWAS that included 58 breast cancer cases, although no association was attained with genome-wide significance level. Among these variants, rs7564590 is in moderate LD with rs13393577 (r^2 ^= 0.44 in CHB+JPT and r^2 ^= 0.25 in CEU) whereas the other two SNPs (rs905883 and rs755861515) are in very weak LD with rs13393577 (all r^2 ^< 0.02 in CHB+JPT and CEU). Thus, if both rs13393577 and rs7564590 are not themselves functional, they might be in high LD with the true causal variants. Additionally, we could not exclude the possibility that the strong association shown in rs13393577 is related to the function of the mir-548f-2 gene harboring SNP rs6956468, which is in tight LD with rs13393577.

## Conclusions

In summary, we have confirmed 10 SNPs in seven loci of breast cancer risk which were initially identified in European or Chinese populations or both and provided additional evidence confirming the heterogeneity in the risk of different tumor subtypes for common breast cancer susceptibility variants. Moreover, we identified rs13393577 in *ERBB4 *located at 2q34 as a new breast cancer susceptibility variant. Future studies, including fine mapping, functional assay, and a replication study with large sample sizes from diverse ethnic populations, are needed to validate our results.

## Abbreviations

CEU: CEPH Utah residents with ancestry from Northern and Western Europe; CHB+JPT: Han Chinese from Beijing + Japanese from Tokyo; CI: confidence interval; ER: estrogen receptor; *ERBB4*: epidermal growth factor receptor 4; GWAS: genome-wide association study; IBS: identity by state; KoGES: Korea Genome Epidemiology Study; LD: linkage disequilibrium; MAF: minor allele frequency; OR: odds ratio; PR: progesterone receptor; QC: quality control; SeBCS: Seoul Breast Cancer Study; SNP: single-nucleotide polymorphism.

## Competing interests

The authors declare that they have no competing interests.

## Authors' contributions

DK helped to conceive and design the experiments and to write the paper. B-GH helped to conceive and design the experiments. JHO, D-JK, MP, E-hK, and W-YP helped to perform the experiments. H-cK and J-YC helped to write the paper. Ji-YL and HS helped to write the paper, to manage the genotyping data, and to perform statistical analyses. Jong-YL coordinated the genetic study. YJK helped to manage the genotyping data and to perform statistical analyses. MJG helped to manage the genotyping data. J-YC and SKP helped to perform statistical analyses and to direct the studies that contributed data or biological collection of original studies. K-ML, YSC, HM, HMK, JP, D-YN, S-HA, K-YY, LL, MHL, S-WK, JWL, B-WP, WH, MKK, S-AL, KM, C-YS, P-EW, C-NH, J-WK, J-PL, S-YJ, and H-LK helped to direct the studies that contributed data or biological collection of original studies. All authors read and approved the final manuscript.

## Supplementary Material

Additional file 1 **Supplementary Methods**. Description of study participants. Genotyping and quality control procedures. Click here for file

Additional file 2 **Supplementary Tables**. Supplementary Table 1. The results and procedure of quality control (QC) in subjects and SNPs on the GWA scan. Supplementary Table 2. Summarized characteristics of study participants and number of SNPs analyzed in each stage. Supplementary Table 3. Per-allele OR and 95% CI for the association of SNPs previously identified and breast cancer risk by ER and PR status in SeBCS. Supplementary Table 4. Summarized results of 17 SNPs included in the Stage II. Supplementary Table 5. The association between the SNPs in flanking region of rs13393577 and breast cancer risk. Click here for file

Additional file 3 **Supplementary Figures**. Supplementary Figure 1. Quantile-quantile (QQ) plot of *p*-values for trend tests of 555,525 SNPs in 2,273 cases and 2,052 controls. Genomic control inflation factor (λ) = 1.043. Supplementary Figure 2. Plot of the first two dimensions from a multidimensional scaling (MDS) analysis based on pairwise identity-by-state (IBS). Gray: Case population in this study, Black: Control population in this study, Blue: HapMap Chinese, Red: HapMap Japanese, Green: HapMap CEU, Purple: HapMap YRI base on the HapMap phase 3. Supplementary Figure 3. Regional plots of the -log *P*-values for 7 SNPs at replicated loci. Results (-log *P*) are shown for the association of directly genotyped and imputed SNPs for a 1 Mb region centered on SNP reported in previous GWAS (diamond). Additional nearby SNP is represented as square. Click here for file
