# Supplementary material for: A genome-wide association study identifies a breast cancer risk variant in ERBB4 at 2q34: results from the Seoul Breast Cancer Study
Source: Breast Cancer Res. 2012 Mar 27;14(2):R56. doi: 10.1186/bcr3158 (PMC3446390; doi:10.1186/bcr3158)
Supplement: Additional file 1 — Supplementary Methods. Description of study participants. Genotyping and quality control procedures. [file bcr3158-S1.DOC]

**Additional file 1: Supplementary Methods**

***Description of study participants***

Seoul Breast Cancer Study (SeBCS)

Breast cancer cases and controls were recruited from the Seoul Breast Cancer Study (SeBCS), a hospital-based case-control study of female breast cancer conducted in Seoul, Korea since 1995. The study has been previously reported elsewhere . Histologically confirmed incident breast cancer cases and controls were recruited in the Seoul National University and ASAN medical center. For Stage I, 2,392 histologically confirmed breast cancer patients were selected for genotyping and 2,052 cases were selected for Stage II among the patients who were not included in Stage I. The study protocol was approved by the Institutional Review Board at Seoul National University Hospital (IRB No. H-0503-144-004).

Korean Hereditary Breast Cancer (KOHBRA)

The KOHBRA study is an ongoing cohort study to examine high risk groups for hereditary breast cancer such as female breast cancer patients with a family history, ovarian cancer, or other coincidental cancers, male breast cancer patients, and family members of breast cancer patients with *BRCA1/2* mutation . We finally selected 1,289 female cancer patients without BRCA1/2 mutation among KOHBRA subjects recruited in 2007-2009. This study has been reviewed and approved by the Institutional Review Board (IRB No. B-0707-047-005).

Yonsei Breast Cancer Study

The Yonsei Breast Cancer Study is a consecutive patient series of 708 female patients who were surgically treated for primary invasive breast cancer in Seoul from 2003 to 2008. This study has been reviewed and approved by the Institutional Review Board of Yonsei University Health System approved this study (4-2009-0483).

Korea Genome Epidemiology Study (KoGES)

Controls were selected from a different sub-project of the Korea Genome Epidemiology Study (KoGES) which is ongoing study since 2001 to investigate major genetic and environmental factors for common diseases in the Korean population. Detailed information has been previously reported . For GWA scan (Stage I), controls were selected from the health examinee cohort (HEXA) from large urban population. It is an ongoing study and a major part of the KoGES. Of 32,887 subjects recruited from 2006 to 2007, 2,392 healthy women with sufficient DNA concentration matched on age (5-year increments) were selected and analyzed in Stage I. For Stage II, controls were selected from another cohort recruited from two small cities with both urban and rural areas aimed to confirm risk factors for diabetes mellitus and hypertension. Of 10,038 subjects surveyed at enrollment in 2001, 2,169 women with sufficient DNA were selected and analyzed. For Stage III, controls were selected from health examinees from rural populations to study the risk factors for chronic diseases. Of the 7,861 subjects recruited from 2005 to 2006, 1,673 women were selected and analyze. This study has been reviewed and approved by the Institutional Review Board (IRB No. 2011-02CON-10②-C) of the Korea National Institute of Health.

***Genotyping and quality control procedures***

GWA scan (Stage I)

*Preparation and quality control of DNA*

Stringent criteria were used to assure data quality. Peripheral whole blood samples for genomic DNA were collected at the time of enrollment of subjects and were visually inspected for adequate fluid volume in individual tubes. The genomic DNA of cases was extracted using the QuickGene DNA whole blood kit S with QuickGene-810 equipment (Fujifilm, Tokyo, Japan). DNA of controls was using G-DEX Genomic DNA extraction kit (iNtRON biotechnology co., Ltd, Korea). All samples were analyzed on a NanoDrop ND-1000 spectrophotometer (Thermo Scientific, USA) for purity, yield, and concentration and on a 1% agarose gel for integrity. Purity was monitored by the A260/A280 and A260/A230 ratios. Of 2,385 cases and 2,392 controls initially, DNA of 5% cases and 14% controls did not reach the condition for GWAS, and finally 2,273 cases and 2,052 controls remained in the GWAS.

*Validation of genotyping platform*

A total of 30 QC samples were genotyped using Affymetrix SNP Array 6.0. The average concordance rate between the QC samples was 99.8%. For internal validation of the Affymetrix SNP array 6.0 platform, 12 SNPs were genotyped for all subjects by SNPstream® UHT (12-plex, SNP-IT assay). To validate possible genotyping errors of GWAS, a concordance test of the main result (rs13393577) was conducted by TaqMan for 3,004 subjects. The concordance rates were 99.7%.

*Exclusion criteria of subjects*

Samples of subjects that had a genotype call rate below 95%, a high heterozygosity rate, or an incorrectly imputed gender were excluded. Calculated genome-wide average IBS between each pair of individuals was used to identify individuals who appeared to be in relationships with first-degree relatives or in relationships with more distant relatives whose clusters were tightly linked to the first-degree relationship. Pairwise IBS between individuals was calculated using a subset of pruned markers (74,965 SNPs) that are in approximate linkage equilibrium. IBS analysis was performed using the PLINK software package. Outliers from multidimensional scaling (MDS) analysis and subjects with past cancer history among controls were also excluded. Finally, 4,325 individuals remained in the association analyses (Additional file 2, Table S1).

*Exclusion criteria of SNPs*

To ensure quality data for SNPs, SNPs were excluded if they met the following quality control criteria: (1) deviation from the Hardy-Weinberg equilibrium *p-*value of <10^-6^, (2) a genotype call rate of <95%, (3) a minor allele frequency of <1% (4) a poor cluster plot for either cases or controls, (5) filtering out differential missingness between cases and controls (*p*<10^-4^), and (6) multiple positioning and/or mitochondrial SNPs. In total, 555,525 Affymetrix SNP Array 6.0 SNPs were used for the final association analyses (Additional file 2, Table S1).

*Quantile-quantile plots*

Distribution of the observed *p-*values of given SNPs was plotted against the theoretical distribution of expected *p-*values to construct quantile-quantile (Q-Q) plots for cases and controls. The genomic inflation factor lamda (λ) was calculated to assess the effect of population stratification (Additional file 3, Figure S1). We did not correct for genomic control in the GWA analyses, as inflation was modest and plots of MAF, Q-Q plot for genotype frequency difference, MDS and PCA suggested that population structure might be disregarded for the samples included in GWA scan.

*Genotyping cluster plots*

Genotype calls for the Affymetrix Genome-wide Human SNP array 6.0 were determined for two separate case and control batches using the Birdseed algorithm. To create a cluster plot for a given SNP, total signal information was processed to generate an integrated summary file. The summary file was then translated into a cluster plot format by a using an algorithm similar to SST 1.0 (SNP signal tool, Affymetrix). Cluster plots were inspected manually for all SNPs considered for the replication studies.

Replication stages (Stage II and Stage III)

*Preparation and quality control of DNA*

Peripheral whole blood samples for genomic DNA were collected at the time of enrollment of subjects. For Stage II, DNA preparation was the same as that for GWAS (Stage I). For Stage III, DNA for cases of KOHBRA study was extracted using the G-DEX Genomic DNA extraction kit (iNtRON biotechnology co., Ltd, Korea). DNA for cases of Yonsei Breast Cancer Study was extracted using the GENEALL biotechnology co., Ltd, Korea kit. DNA for controls was prepared by the same procedure to Stage I. All samples were monitored by the same standard QC method of Stage I. Genotyping for Stage II and Stage III was conducted under the standard protocol at the Center for Genome Science of the Korea National Institute of Health. We obtained a high concordance rate of 97% among 61 pairs of blind duplicates with an average call rate of 98.7%.

**Supplementary References**

1. Park SK, Yoo KY, Lee SJ, Kim SU, Ahn SE, Noh DY, Choe KJ, Strickland PT, Hirvonen A, Kang DH: **Alcohol consumption, glutathione S-transferase M1 and T1 genetic polymorphisms and breast cancer risk.** *Pharmacogenetics* 2000, **10:**301-309.

2. Choi JY, Lee KM, Park SK, Noh DY, Ahn SH, Yoo KY, Kang DH: **Association of paternal age at birth and the risk of breast cancer in offspring: a case control study.** *Bmc Cancer* 2005, **5**.

3. Han SH, Lee KM, Choi JY, Park SK, Lee JY, Lee JE, Noh DY, Ahn SH, Han WS, Kim DH, Hong YC, Ha E, Yoo KY, Kang DH: **CASP8 polymorphisms, estrogen and progesterone receptor status, and breast cancer risk.** *Breast Cancer Res Tr* 2008, **110:**387-393.

4. Han SA, Park SK, Ahn SH, Lee MH, Noh DY, Kim LS, Noh WC, Jung Y, Kim KS, Kim SW, Grp KBCS: **The Korean Hereditary Breast Cancer (KOHBRA) Study: Protocols and Interim Report.** *Clin Oncol-Uk* 2011, **23:**434-441.

5. Kim YJ, Go MJ, Hu C, Hong CB, Kim YK, Lee JY, Hwang JY, Oh JH, Kim DJ, Kim NH, Kim S, Hong EJ, Kim JH, Min H, Kim Y, Zhang R, Jia WP, Okada Y, Takahashi A, Kubo M, Tanaka T, Kamatani N, Matsuda K, Park T, Oh B, Kimm K, Kang D, Shin C, Cho NH, Kim HL, et al: **Large-scale genome-wide association studies in east Asians identify new genetic loci influencing metabolic traits.** *Nat Genet* 2011, **43:**990-U102.

6. Cho YS, Go MJ, Kim YJ, Heo JY, Oh JH, Ban HJ, Yoon D, Lee MH, Kim DJ, Park M, Cha SH, Kim JW, Han BG, Min H, Ahn Y, Park MS, Han HR, Jang HY, Cho EY, Lee JE, Cho NH, Shin C, Park T, Park JW, Lee JK, Cardon L, Clarke G, McCarthy MI, Lee JY, Lee JK, et al: **A large-scale genome-wide association study of Asian populations uncovers genetic factors influencing eight quantitative traits.** *Nat Genet* 2009, **41:**527-534.
