# Supplementary material for: A genome-wide association study identifies a breast cancer risk variant in ERBB4 at 2q34: results from the Seoul Breast Cancer Study
Source: Breast Cancer Res. 2012 Mar 27;14(2):R56. doi: 10.1186/bcr3158 (PMC3446390; doi:10.1186/bcr3158)
Supplement: Additional file 2 — Supplementary Tables. Supplementary Table 1. The results and procedure of quality control (QC) in subjects and SNPs on the GWA scan. Supplementary Table 2. Summarized characteristics of study participants and number of SNPs analyzed in each stage. Supplementary Table 3. Per-allele OR and 95% CI for the association of SNPs previously identified and breast cancer risk by ER and PR status in SeBCS. Supplementary Table 4. Summarized results of 17 SNPs included in the Stage II. Supplementary Table 5. The association between the SNPs in flanking region of rs13393577 and breast cancer risk. [file bcr3158-S2.DOC]

**Additional file 2: Supplementary Tables**

| Table S1. The results and procedure of quality control (QC) in subjects and SNPs on the GWA scan | | | |
| --- | --- | --- | --- |
|  | N of cases or  SNPs in case group | | N of controls or  SNPs in control group |
| QC procedure in subjects |  | |  |
| Subjects at the start of QC | 2,385 | | 2,392 |
| Exclusion of subjects by |  | |  |
| Gender inconsistency | 0 | | 1 |
| Call rate (<95%) | 17 | | 252 |
| Excessive heterozygosity | 12 | | 15 |
| IBS relatives | 14 | | 16 |
| MDS outliers | 0 | | 11 |
| Past history of cancer | 40 | | 45 |
| Benign breast cancer | 29 | |  |
| Final subjects | 2,273 | | 2,052 |
| QC in SNPs |  | |  |
| SNPs at the start of QC in each group | 909,622 | | 909,622 |
| Exclusion of SNPs by |  | |  |
| HWE < 10^-6^ |  | | 17,697 |
| Call rate < 95% | 50,561^a^ | | 94,694^a^ |
| MAF < 0.01 | 210,952^a^ | | 182,960^a^ |
| Common SNPs | 631,882 | | |
| Exclusion by |  |  | |
| Differential missingness <10^-4^ | 62,534 | | |
| Multiple position & mitochondrial SNPs | 13,823 | | |
| Final SNPs in total subjects | 555,525 | | |

^a^ Not mutually exclusive

| Table S2. Summarized characteristics of study participants and number of SNPs analyzed in each stage | | | | | |
| --- | --- | --- | --- | --- | --- |
| Study population | Cases | |  | Controls | |
|  | N | Age^b^ |  | N | Age^b^ |
| Stage I (555,525 SNPs)^a^ | 2,273 | 47.3 (9.8) |  | 2,052 | 51.6 (7.6) |
| Stage II (17 SNPs) | 2,052 | 46.6 (10.1) |  | 2,169 | 51.3 (8.9) |
| Stage III (4 SNPs) | 1,997 | 44.2 (10.2) |  | 1,676 | 49.0 (6.9) |

^a^ Selected from SNPs included in the Affymetrix 6.0 SNP array with MAF≥1%, Call rate≥95%, and QC consistency, ^b^ Mean (SD).

| Table S3. Per-allele OR and 95% CI for the association of SNPs previously identified and breast cancer risk by ER and PR status in SeBCS. | | | | | | | | | | | | | | |
| --- | --- | --- | --- | --- | --- | --- | --- | --- | --- | --- | --- | --- | --- | --- |
| Chr | SNP | ER-positive (n=1,307) | |  | ER-negative (n=735) | | Case-only *P*^b^ |  | PR-positive (n=1,127) | |  | PR-negative (n=903) | | Case-only *P*^b^ |
|  |  | OR (95% CI) ^a^ | *p*-value |  | OR (95% CI) ^a^ | *p*-value |  |  | OR (95% CI) ^a^ | *p*-value |  | OR (95% CI) ^a^ | *p*-value |  |
| 1p11.2 | rs11249433 | 1.10 (0.84-1.43) | 0.496 |  | 1.20 (0.89-1.61) | 0.225 | 0.295 |  | 1.12 (0.84-1.49) | 0.444 |  | 1.18 (0.90-1.55) | 0.228 | 0.302 |
| 2q35 | rs13387042 | 1.19 (1.02-1.40) | 0.030 |  | 1.04 (0.86-1.25) | 0.722 | 0.085 |  | 1.18 (1.00-1.40) | 0.049 |  | 1.07 (0.90-1.28) | 0.426 | 0.169 |
| 3p24.1 | rs4973768 | 1.13 (1.00-1.27) | 0.048 |  | 1.03 (0.89-1.18) | 0.721 | 0.246 |  | 1.09 (0.96-1.24) | 0.183 |  | 1.08 (0.95-1.23) | 0.228 | 0.967 |
| 5q11.2 | rs16886165 | 1.16 (1.05-1.29) | 0.004 |  | 1.11 (0.98-1.25) | 0.089 | 0.470 |  | 1.13 (1.01-1.27) | 0.027 |  | 1.14 (1.02-1.27) | 0.019 | 0.876 |
|  | rs889312 | 1.15 (1.04-1.27) | 0.006 |  | 1.18 (1.05-1.32) | 0.004 | 0.663 |  | 1.14 (1.02-1.26) | 0.019 |  | 1.18 (1.07-1.32) | 0.002 | 0.466 |
| 5p12 | rs7716600 | 1.20 (1.09-1.33) | 3.5 x 10^-4^ |  | 1.03 (0.92-1.16) | 0.573 | 0.019 |  | 1.16 (1.04-1.29) | 0.006 |  | 1.10 (0.99-1.23) | 0.067 | 0.731 |
|  | rs4415084 | 1.13 (1.02-1.26) | 0.017 |  | 1.00 (0.89-1.13) | 0.982 | 0.048 |  | 1.10 (0.99-1.23) | 0.079 |  | 1.05 (0.94-1.17) | 0.371 | 0.529 |
| 5p15.2 | rs1092913 | 1.15 (1.03-1.28) | 0.010 |  | 1.05 (0.93-1.19) | 0.399 | 0.121 |  | 1.12 (1.01-1.26) | 0.041 |  | 1.09 (0.98-1.22) | 0.121 | 0.373 |
| 6q22.33 | rs2180341 | 1.01 (0.90-1.12) | 0.925 |  | 1.04 (0.92-1.18) | 0.532 | 0.587 |  | 1.03 (0.91-1.15) | 0.679 |  | 1.01 (0.90-1.14) | 0.871 | 0.652 |
| 6q25.1 | rs3734805 | 1.16 (1.04-1.29) | 0.008 |  | 1.29 (1.14-1.46) | 4.8 x 10^-5^ | 0.145 |  | 1.19 (1.06-1.34) | 0.002 |  | 1.22 (1.09-1.37) | 6.0 x 10^-4^ | 0.826 |
|  | rs2046210 | 1.26 (1.13-1.39) | 1.2 x 10^-5^ |  | 1.35 (1.20-1.51) | 5.0 x 10^-7^ | 0.413 |  | 1.28 (1.15-1.43) | 5.1 x 10^-6^ |  | 1.29 (1.16-1.44) | 2.9 x 10^-6^ | 0.997 |
| 7q32.3 | rs2048672 | 1.06 (0.96-1.17) | 0.230 |  | 1.03 (0.92-1.16) | 0.585 | 0.653 |  | 1.02 (0.92-1.13) | 0.762 |  | 1.08 (0.98-1.20) | 0.132 | 0.250 |
| 8q24.21 | rs13281615 | 1.05 (0.95-1.16) | 0.341 |  | 1.01 (0.90-1.13) | 0.859 | 0.541 |  | 1.02 (0.92-1.13) | 0.732 |  | 1.04 (0.94-1.16) | 0.435 | 0.726 |
|  | rs1562430 | 1.14 (0.97-1.32) | 0.103 |  | 1.15 (0.97-1.37) | 0.118 | 0.846 |  | 1.14 (0.97-1.34) | 0.113 |  | 1.14 (0.97-1.35) | 0.110 | 0.747 |
| 9p21.3 | rs1011970 | 1.00 (0.83-1.22) | 0.971 |  | 0.99 (0.79-1.24) | 0.916 | 0.796 |  | 1.07 (0.87-1.31) | 0.530 |  | 0.93 (0.76-1.15) | 0.530 | 0.248 |
| 9q31.2 | rs865686 | 0.93 (0.77-1.13) | 0.466 |  | 1.18 (0.94-1.49) | 0.151 | 0.075 |  | 0.94 (0.77-1.15) | 0.551 |  | 1.09 (0.89-1.35) | 0.403 | 0.476 |
| 10p15.1 | rs2380205 | 1.20 (1.01-1.43) | 0.037 |  | 0.95 (0.78-1.14) | 0.568 | 0.030 |  | 1.20 (1.00-1.44) | 0.053 |  | 0.98 (0.82-1.17) | 0.836 | 0.040 |
| 10q21.2 | rs10995190 | 0.89 (0.64-1.24) | 0.490 |  | 1.40 (0.91-2.15) | 0.131 | 0.050 |  | 0.92 (0.65-1.30) | 0.622 |  | 1.20 (0.82-1.76) | 0.353 | 0.146 |
|  | rs10822013 | 1.09 (0.99-1.20) | 0.089 |  | 1.04 (0.93-1.17) | 0.485 | 0.542 |  | 1.10 (0.99-1.22) | 0.076 |  | 1.04 (0.94-1.15) | 0.470 | 0.685 |
| 10q22.3 | rs704010 | 1.05 (0.95-1.17) | 0.337 |  | 1.08 (0.96-1.23) | 0.205 | 0.306 |  | 1.02 (0.91-1.15) | 0.718 |  | 1.12 (1.00-1.25) | 0.051 | 0.081 |
| 10q26.13 | rs10510102 | 0.99 (0.87-1.12) | 0.853 |  | 1.07 (0.93-1.24) | 0.328 | 0.168 |  | 1.03 (0.90-1.19) | 0.629 |  | 1.03 (0.90-1.17) | 0.723 | 0.741 |
|  | rs10736303^c^ | 1.14 (1.04 - 1.26) | 0.008 |  | 1.16 (1.30-1.04) | 0.008 | 0.612 |  | 1.17 (1.30-1.06) | 0.002 |  | 1.17 (1.04-1.32) | 0.029 | 0.381 |
| 11p15.5 | rs3817198 | 0.96 (0.84-1.10) | 0.584 |  | 1.08 (0.92-1.27) | 0.332 | 0.118 |  | 0.95 (0.82-1.09) | 0.453 |  | 1.08 (0.93-1.24) | 0.324 | 0.093 |
| 14q24.1 | rs10483813^d^ | 1.14 (0.85-1.53) | 0.367 |  | 1.30 (0.92-1.84) | 0.139 | 0.570 |  | 1.17 (0.85-1.60) | 0.327 |  | 1.22 (0.89-1.67) | 0.214 | 0.855 |
| 16q12.1 | rs4784227 | 1.25 (1.12-1.39) | 5.9 x 10^-5^ |  | 1.27 (1.13-1.44) | 1.2 x 10^-4^ | 0.961 |  | 1.27 (1.13-1.42) | 4.6 x 10^-5^ |  | 1.25 (1.12-1.40) | 1.2 x 10^-4^ | 0.492 |
|  | rs3803662 | 1.24 (1.12-1.37) | 3.7 x 10^-5^ |  | 1.25 (1.11-1.41) | 1.7 x 10^-4^ | 0.889 |  | 1.28 (1.15-1.43) | 1.1 x 10^-5^ |  | 1.22 (1.09-1.36) | 3.2 x 10^-4^ | 0.468 |
| 19q13.41 | rs10411161 | 1.10 (0.98-1.23) | 0.093 |  | 0.90 (0.80-1.02) | 0.111 | 0.004 |  | 1.08 (0.96-1.22) | 0.197 |  | 0.96 (0.85-1.07) | 0.431 | 0.047 |
| Abbreviations: Chr, chromosome; SNP, single nucleotide polymorphism; ER, estrogen receptor; PR, progesterone receptor; OR, odds ratio; CI, confidence interval  ^a^ Per-allele OR adjusted for age; ^b^ Case-only P-value was used to test for heterogeneity, and was estimated using a polytomous logistic regression model with receptor status as the outcome; ^c^ Proxy SNP of rs2981579; ^d^ Proxy SNP of rs999797 | | | | | | | | | | | | | | |

| Table S4. Summarized results for 17 SNPs included in the Stage II | | | | | | | | | | | | | |
| --- | --- | --- | --- | --- | --- | --- | --- | --- | --- | --- | --- | --- | --- |
| SNP | Genomic location^a^  (chromosomal location) | Gene | Stage I | | | |  |  | | Stage II | | | |
|  |  |  | MAF | | OR (95% CI)^b^ | *p*-value |  | MAF | | | OR (95% CI)^b^ | *p*-value | FDR *p* |
|  |  |  | Cases | Controls |  |  |  | Cases | Controls | |  |  |  |
| rs13393577 | 213,005,108 (2q34) | *ERBB4* | 0.07 | 0.04 | 1.69 (1.38-2.06) | 4.8 x 10^-7^ |  | 0.10 | 0.07 | | 1.51 (1.29-1.78) | 7.2 x 10^-7^ | 1.1 x 10^-5^ |
| rs9498283 | 149,646,875(6q25.1) | *MAP3K7IP2* | 0.45 | 0.50 | 0.81 (0.75-0.89) | 5.3 x 10^-6^ |  | 0.45 | 0.47 | | 0.92 (0.85-1.01) | 8.0 x 10^-2^ | 6.8 x 10^-1^ |
| rs11077488 | 65,801,677(17q24.3) | *KCNJ2* | 0.15 | 0.19 | 0.77 (0.69-0.87) | 1.2 x 10^-5^ |  | 0.16 | 0.17 | | 0.90 (0.80-1.01) | 7.2 x 10^-2^ | 9.7 x 10^-1^ |
| rs3806685 | 180,522,445 (3q26.32) | *ZNF639* | 0.16 | 0.19 | 0.81 (0.74-0.89) | 2.4 x 10^-6^ |  | 0.17 | 0.18 | | 0.90 (0.80-1.01) | 7.7 x 10^-2^ | 9.7 x 10^-1^ |
| rs1953290 | 81,015,533 (13q31.1) | *Intergenic* | 0.15 | 0.19 | 0.72 (0.64-0.81) | 4.8 x 10^-8^ |  | 0.17 | 0.16 | | 1.06 (0.94-1.19) | 3.6 x 10^-1^ | 9.7 x 10^-1^ |
| rs10517055 | 42,597,491 (4p13) | *LOC389207* | 0.10 | 0.14 | 0.72 (0.62-0.82) | 1.8 x 10^-6^ |  | 0.13 | 0.12 | | 1.12 (0.99-1.27) | 8.5 x 10^-1^ | 9.7 x 10^-1^ |
| rs12487595 | 190,001,128 (3q28) | *LPP* | 0.08 | 0.06 | 1.51 (1.26-1.81) | 8.8 x 10^-6^ |  | 0.08 | 0.08 | | 1.00 (0.84-1.18) | 9.7 x 10^-1^ | 9.7 x 10^-1^ |
| rs10814071 | 34,136,645 (9p13.3) | *Intergenic* | 0.18 | 0.22 | 0.79(0.71-0.88) | 2.3 x 10^-5^ |  | 0.19 | 0.19 | | 0.98 (0.88-1.10) | 7.7 x 10^-1^ | 9.7 x 10^-1^ |
| rs3803662 | 51,143,842 (16q12.1) | *LOC643714* | 0.33 | 0.38 | 0.78 (0.69-0.88) | 3.4 x 10^-5^ |  | 0.35 | 0.36 | | 0.96 (0.87-1.05) | 3.7 x 10^-1^ | 9.7 x 10^-1^ |
| rs10251934 | 144,333,510 (7q35) | *tcag7.926* | 0.25 | 0.21 | 1.25(1.13-1.39) | 2.5 x 10^-5^ |  | 0.24 | 0.24 | | 0.99 (0.89-1.10) | 8.1 x 10^-1^ | 9.7 x 10^-1^ |
| rs9320374 | 112,090,511 (6q21) | *FYN* | 0.42 | 0.38 | 1.21(1.11-1.33) | 2.9 x 10^-5^ |  | 0.41 | 0.40 | | 1.04 (0.94-1.14) | 4.6 x 10^-1^ | 9.7 x 10^-1^ |
| rs2597540 | 121,965,682 (4q27) | *PRDM5* | 0.41 | 0.45 | 0.82(0.75-0.90) | 4.1 x 10^-5^ |  | 0.43 | 0.43 | | 1.02 (0.93-1.12) | 6.5 x 10^-1^ | 9.7 x 10^-1^ |
| rs9436636 | 61,585,361 (1p31.3) | *NFIA* | 0.31 | 0.35 | 0.83(0.75-0.91) | 5.3 x 10^-5^ |  | 0.33 | 0.34 | | 0.96 (0.88-1.06) | 4.5 x 10^-1^ | 9.7 x 10^-1^ |
| rs17164117 | 91,245,705 (7q21.2) | *Intergenic* | 0.33 | 0.37 | 0.84(0.77-0.92) | 1.1 x 10^-4^ |  | 0.35 | 0.34 | | 1.01 (0.92-1.11) | 8.6 x 10^-1^ | 9.7 x 10^-1^ |
| rs7580896 | 180,791,941 (2q31.3) | *Intergenic* | 0.17 | 0.20 | 0.80 (0.72-0.90) | 1.3 x 10^-4^ |  | 0.18 | 0.18 | | 1.01 (0.90-1.13) | 8.6 x 10^-1^ | 9.7 x 10^-1^ |
| rs2758862 | 39,347,100 (6p21.2) | *Intergenic* | 0.35 | 0.39 | 0.84(0.76-0.92) | 1.1 x 10^-4^ |  | 0.40 | 0.41 | | 0.96 (0.87-1.05) | 3.4 x 10^-1^ | 9.7 x 10^-1^ |
| rs558366 | 79,904,872 (13q31.1) | *Intergenic* | 0.41 | 0.37 | 1.18 (1.08-1.30) | 2.5 x 10^-4^ |  | 0.38 | 0.38 | | 1.00 (0.92-1.10) | 9.4 x 10^-1^ | 9.7 x 10^-1^ |
| Abbreviations: SNP, single nucleotide polymorphism; MAF, minor allele frequency; OR, odds ratio; CI, confidence interval  ^a^ Location is based on NCBI Build 36; ^b^Per-allele OR adjusted for age | | | | | | | | | | | | | |

Table S5. The association between the SNPs in flanking region of rs13393577 and breast cancer risk.

| SNP | CHR | Genomic location | r^2^ | Major/  Minor allele | MAF^a^ | Pre-allele OR^b^ | *P*-trend | genotype |
| --- | --- | --- | --- | --- | --- | --- | --- | --- |
| rs13393577 | 2 | 213005108 |  | C/T | 0.053 | 1.69 (1.38-2.06) | 4.8E-07 | typed |
| rs6756468 | 2 | 212983576 | 1 | C/T | 0.057 | 1.38 (1.14-1.67) | 1.2E-03 | typed |
| rs10188926 | 2 | 212983549 | 1 | A/G | 0.059 | 1.27 (1.05-1.52) | 1.3E-02 | imputed |
| rs6712295 | 2 | 212983358 | 1 | G/A | 0.059 | 1.27 (1.05-1.52) | 1.3E-02 | imputed |
| rs13388956 | 2 | 212988683 | 1 | C/T | 0.059 | 1.26 (1.05-1.52) | 1.3E-02 | imputed |
| rs13400881 | 2 | 212988749 | 1 | C/A | 0.059 | 1.26 (1.05-1.52) | 1.3E-02 | imputed |
| rs10167244 | 2 | 212989555 | 1 | G/A | 0.059 | 1.26 (1.05-1.52) | 1.3E-02 | imputed |
| rs10194540 | 2 | 212990609 | 1 | C/G | 0.059 | 1.26 (1.05-1.52) | 1.3E-02 | imputed |
| rs13417523 | 2 | 212991024 | 1 | A/G | 0.059 | 1.26 (1.05-1.52) | 1.3E-02 | imputed |
| rs1394780 | 2 | 212991435 | 1 | G/T | 0.059 | 1.26 (1.05-1.52) | 1.4E-02 | imputed |
| rs10202760 | 2 | 212999599 | 0.83 | C/T | 0.059 | 1.26 (1.05-1.52) | 1.4E-02 | imputed |
| rs13383863 | 2 | 212992266 | 1 | G/T | 0.059 | 1.26 (1.05-1.52) | 1.4E-02 | imputed |
| rs13413335 | 2 | 212996909 | 1 | C/A | 0.059 | 1.26 (1.05-1.52) | 1.4E-02 | imputed |
| rs16848753 | 2 | 213098009 | 0.81 | G/A | 0.065 | 1.23 (1.03-1.47) | 2.6E-02 | typed |

^a^MAF in all subjects

^b^Per-allele OR adjusted for age
