# Supplementary material for: A genome-wide association study identifies a breast cancer risk variant in ERBB4 at 2q34: results from the Seoul Breast Cancer Study
Source: Breast Cancer Res. 2012 Mar 27;14(2):R56. doi: 10.1186/bcr3158 (PMC3446390; doi:10.1186/bcr3158)
Supplement: Additional file 3 — Supplementary Figures. Supplementary Figure 1. Quantile-quantile (QQ) plot of p-values for trend tests of 555,525 SNPs in 2,273 cases and 2,052 controls. Genomic control inflation factor (λ) = 1.043. Supplementary Figure 2. Plot of the first two dimensions from a multidimensional scaling (MDS) analysis based on pairwise identity-by-state (IBS). Gray: Case population in this study, Black: Control population in this study, Blue: HapMap Chinese, Red: HapMap Japanese, Green: HapMap CEU, Purple: HapMap YRI base on the HapMap phase 3. Supplementary Figure 3. Regional plots of the -log P-values for 7 SNPs at replicated loci. Results (-log P) are shown for the association of directly genotyped and imputed SNPs for a 1 Mb region centered on SNP reported in previous GWAS (diamond). Additional nearby SNP is represented as square. [file bcr3158-S3.DOC]

**Additional file 3: Supplementary Figures**

Figure S1. Quantile-quantile (QQ) plot of *p*-values in 2,273 cases and 2,052 controls.


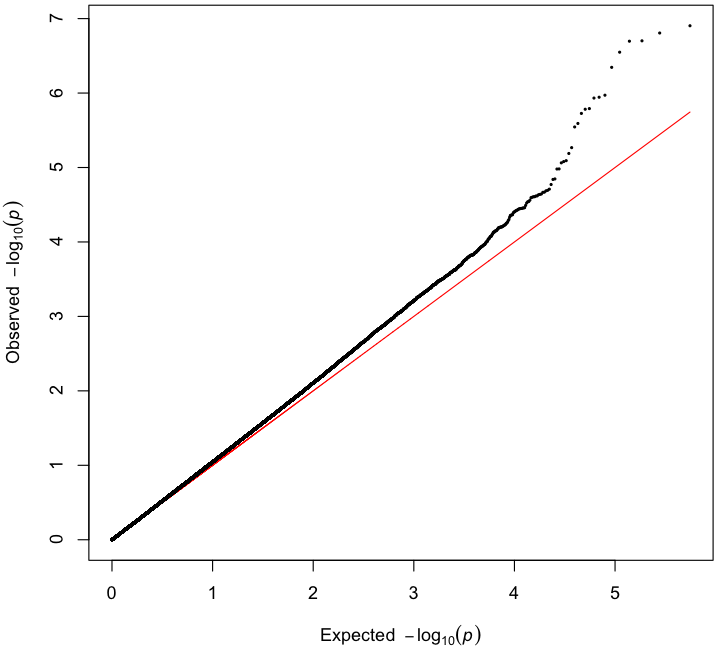


**λ= 1.043**

Figure S2: Plot of the first two dimensions from a multidimensional scaling (MDS) analysis based on pairwise identity-by-state (IBS).


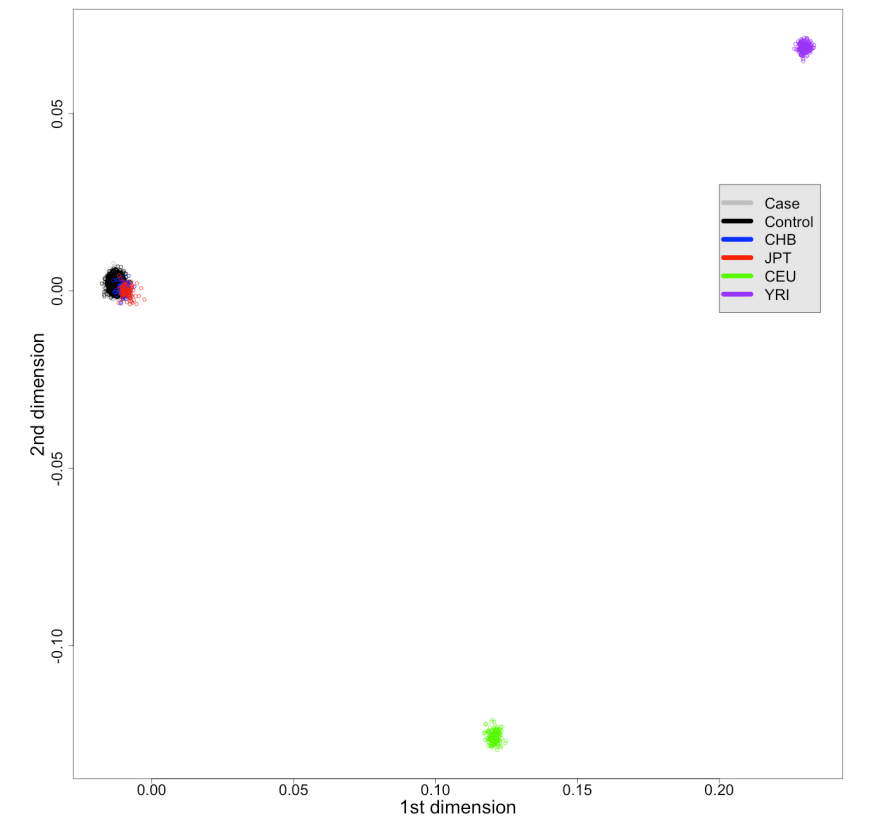


Gray: Case population in this study, Black: Control population in this study, Blue: HapMap Chinese, Red: HapMap Japanese, Green: HapMap CEU, Purple: HapMap YRI base on the HapMap phase 3.

Figure S3: Regional plots of the −log *P*-values for 7 SNPs at replicated loci. Results (−log *P*) are shown for the association of directly genotyped and imputed SNPs for a 1 Mb region centered on SNP reported in previous GWAS (diamond). Additional nearby SNP is represented as square.

[rs889312 and rs16886165 at 5q11.2]

[rs7716600 at 5q12]

[rs1092913 at 5p15.2]

[rs2046210 and rs3734805 at 6q25.1]

[rs1562430 at 8q24.21]

[rs10736303 at 10q26.13]

[rs4784227 and rs3803662 at 16q12.1]
